# Canagliflozin Mitigates Diabetic Cardiomyopathy through Enhanced PINK1-Parkin Mitophagy

**DOI:** 10.3390/ijms25137008

**Published:** 2024-06-26

**Authors:** Chunru Yang, Cheng Xiao, Zerui Ding, Xiaojun Zhai, Jieying Liu, Miao Yu

**Affiliations:** 1 Key Laboratory of Endocrinology National Health Commission, Department of Endocrinology, Peking Union Medical College Hospital, Chinese Academy of Medical Sciences & Peking Union Medical College, Beijing 100730, China; pumc_yangcr@student.pumc.edu.cn (C.Y.);; 2 Center for Biomarker Discovery and Validation, National Infrastructures for Translational Medicine (PUMCH), Institute of Clinical Medicine, Peking Union Medical College Hospital, Chinese Academy of Medical Sciences & Peking Union Medical College, Beijing 100730, China

**Keywords:** diabetic cardiomyopathy, mitophagy, mitochondrial dysfunction, mitochondrial biogenesis, sodium-glucose co-transporter-2 inhibitor

## Abstract

Diabetic cardiomyopathy (DCM) is a major determinant of mortality in diabetic populations, and the potential strategies are insufficient. Canagliflozin has emerged as a potential cardioprotective agent in diabetes, yet its underlying molecular mechanisms remain unclear. We employed a high-glucose challenge (60 mM for 48 h) in vitro to rat cardiomyocytes (H9C2), with or without canagliflozin treatment (20 µM). In vivo, male C57BL/6J mice were subjected to streptozotocin and a high-fat diet to induce diabetes, followed by canagliflozin administration (10, 30 mg·kg^−1^·d^−1^) for 12 weeks. Proteomics and echocardiography were used to assess the heart. Histopathological alterations were assessed by the use of Oil Red O and Masson’s trichrome staining. Additionally, mitochondrial morphology and mitophagy were analyzed through biochemical and imaging techniques. A proteomic analysis highlighted alterations in mitochondrial and autophagy-related proteins after the treatment with canagliflozin. Diabetic conditions impaired mitochondrial respiration and ATP production, alongside decreasing the related expression of the PINK1-Parkin pathway. High-glucose conditions also reduced PGC-1α-TFAM signaling, which is responsible for mitochondrial biogenesis. Canagliflozin significantly alleviated cardiac dysfunction and improved mitochondrial function both in vitro and in vivo. Specifically, canagliflozin suppressed mitochondrial oxidative stress, enhancing ATP levels and sustaining mitochondrial respiratory capacity. It activated PINK1-Parkin-dependent mitophagy and improved mitochondrial function via increased phosphorylation of adenosine monophosphate-activated protein kinase (AMPK). Notably, PINK1 knockdown negated the beneficial effects of canagliflozin on mitochondrial integrity, underscoring the critical role of PINK1 in mediating these protective effects. Canagliflozin fosters PINK1-Parkin mitophagy and mitochondrial function, highlighting its potential as an effective treatment for DCM.

## 1. Introduction

The number of individuals with diabetes mellitus is increasing globally. The International Diabetes Federation estimates that by 2045, 800 million people worldwide will be affected by diabetes and its complications [1]. Diabetic cardiomyopathy (DCM), a primary complication of diabetes mellitus, occurs independently of coronary artery disease and hypertension [2]. DCM hallmarks involve abnormal cardiac function and structure, including hypertrophy, interstitial fibrosis, cardiomyocyte apoptosis, and diastolic dysfunction [3]. Despite its prevalence, therapeutic options for DCM remain limited.

Mitochondrial functionality is pivotal for cardiac health, as the heart, a high-energy organ, relies on mitochondrial adenosine triphosphate (ATP) for efficient operation [4]. Accumulating evidence indicates mitochondrial dysfunction in the pathogenesis of DCM [5], yet the molecular underpinnings remain inadequately defined.

Mitophagy refers to the specific removal of damaged, aging, and reactive oxygen species (ROS)-excess mitochondria through the autophagic lysosome pathway, promoting their regeneration and recycling, and maintaining the number of mitochondria in the cell to ensure the energy supply and normal function of the cell [6,7]. The PTEN-induced kinase 1 (PINK1)-Parkin-dependent pathway, one of the classical mechanisms of mitophagy, is critical for removing dysfunctional mitochondria following damage [8,9]. A decrease in membrane potential prevents PINK1 from entering the inner membrane, leading to its accumulation on the outer membrane. Then, PINK1 undergoes dimerization and becomes activated through autophosphorylation. Subsequently, the activated PINK1 recruits Parkin to initiate mitophagy [10,11,12]. Diminished mitophagy has been observed in the context of diabetes-related myocardial injury, and enhancing mitophagy has shown promise in improving mitochondrial function and alleviating DCM [13,14]. Therefore, identifying a safe and effective pharmacotherapy to promote mitophagy has significant implications for managing DCM.

Canagliflozin, a novel oral hypoglycemic agent within the sodium-glucose co-transporter 2 inhibitor (SGLT2i) class, modulates renal tubular function to diminish urinary glucose reabsorption, effectively lowering blood glucose levels [15]. Demonstrated through extensive clinical trials, canagliflozin significantly reduces the risk of cardiovascular mortality and hospitalization in patients with heart failure (HF) and type 2 diabetes mellitus, underscoring its cardioprotective properties [16,17,18]. But the mechanism is unknown. Empagliflozin, another SGLT2i, has been recognized as attenuating cardiac ischemia/reperfusion or diabetic heart through activating the receptor-dependent mitophagy pathway [19,20]. Therefore, SGLT2i may be an effective mitochondrial autophagy agonist. This led us to explore whether other SGLT2i, particularly canagliflozin, which has received less attention, could exert their protective effects on diabetic cardiomyopathy through enhancing mitophagy, especially the PINK1-Parkin-dependent pathway.

Hence, this investigation aimed to explore the impacts of canagliflozin and its mechanism in both diabetic hearts and hyperglycemia-exposed cardiomyocytes in vitro. The focus was specifically on unraveling the molecular mechanisms that underpin the regulation of mitophagy by canagliflozin. By delving into these areas, we sought to extend the understanding of how canagliflozin potentially contributes to a cardioprotective effect, particularly through the modulation of mitochondrial quality control pathways.

## 2. Results

### 2.1. Canagliflozin Improved Mitochondrial Quality in High-Glucose-Treated H9C2 Cells

**Figure 1:**
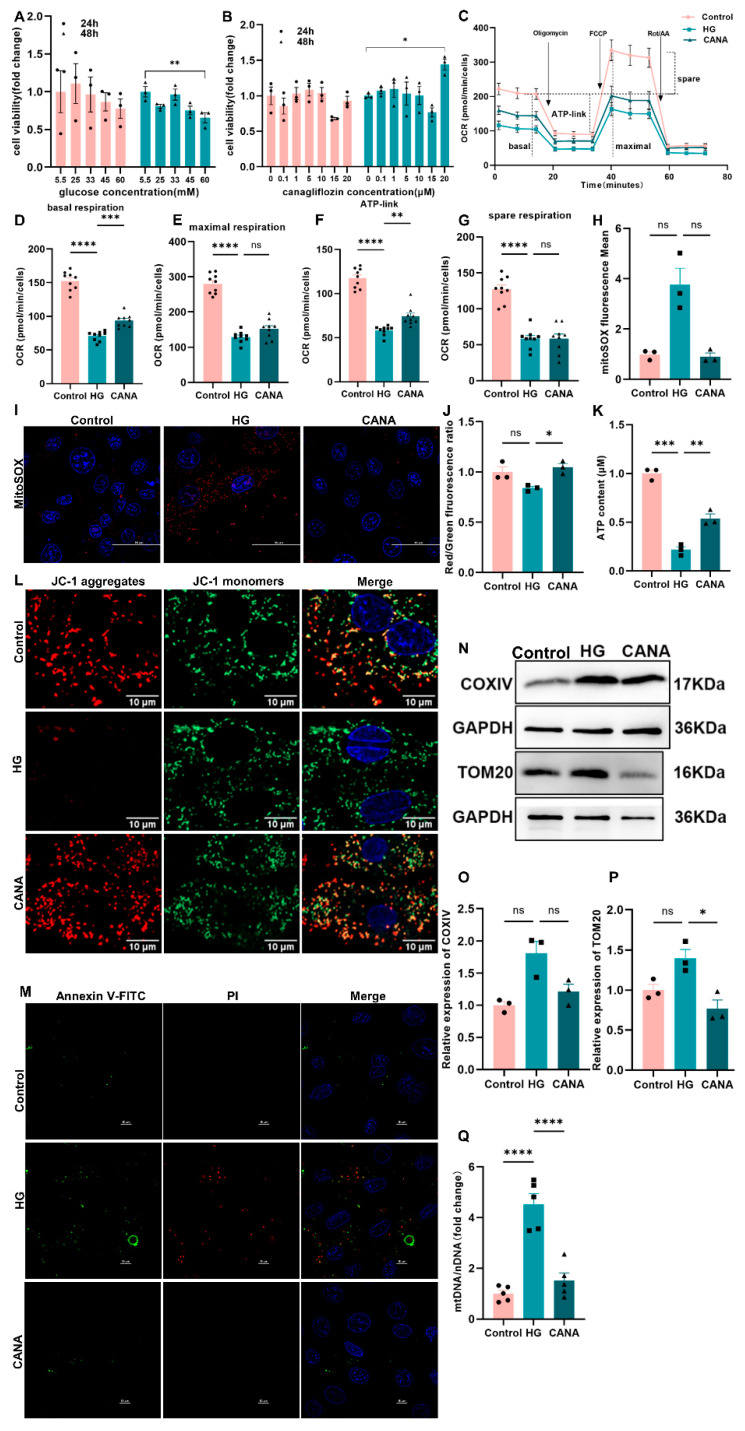
Canagliflozin improved mitochondrial quality in high-glucose-treated H9C2 cells. (**A**) Cell viability Cell Counting Kit-8 (CCK8) assay, different glucose concentrations were intervened for 48 h, *n* = 3. (**B**) CCK8 assay, cells were treated with high glucose (60 mM) and none or different concentrations of canagliflozin, including 0.1 μM, 1 μM, 5 μM, 10 μM, 15 μM, and 20 μM for 12 or 48 h. *n* = 3. (**C**) Schematic of oxygen consumption rate of H9C2 cells during the Seahorse assay (normalized OCR). (**D**–**G**) The four main parameters of Seahorse assay are as follows: (1) basal respiration; (2) maximal respiratory capacity; (3) adenosine triphosphate production; and (4) spare respiratory. (**H**) Relative mean fluorescence intensity of MitoSOX (Mean = IntDen/Area). (**I**) Mitochondrial reactive oxygen species detection using MitoSOX (red); scale bar: 50 μm; original magnification: ×100. (**J**) Quantification of mitochondrial membrane potential changes (JC-1 aggregates/JC-1 monomer). (**K**) ATP content determination in H9C2 cells (*n* = 3) using an ATP assay kit. (**L**) Measurement of mitochondrial membrane potential using JC-1 staining; scale bar: 10 μm; original magnification: ×100. (**M**) Cell apoptosis was examined through Annexin V-FITC/PI staining; scale bar: 10 μm; original magnification: ×100. (**N**–**P**) Western blot for COXIV and TOM20 proteins; all protein expressions were normalized to GAPDH protein expression. (**Q**) The ratio of mitochondrial-encoded DNA (mtDNA) to nuclear-encoded DNA (nDNA), *n* = 5. Control, 5.5 mmol/L glucose; HG, 60 mmol/L glucose; CANA, HG + 20 μmol/L canagliflozin. All data are shown as mean ± SEM. * *p* < 0.05; ** *p* < 0.01; *** *p* < 0.001; and **** *p* < 0.0001; ns, no significance.

Initial assessments utilizing Cell Counting Kit-8 indicated that exposure to a high glucose concentration (60 mM) for 48 h significantly reduced cell viability (Figure 1A). Conversely, treatment with canagliflozin (20 μM) for the same duration ameliorated the detrimental effects of high glucose on cell viability (Figure 1B), establishing the optimal conditions for the glucose and drug concentrations and intervention duration. The mitochondrial stress test further delineated the impact on mitochondrial function, revealing that high glucose (HG) exposure markedly impaired basal mitochondrial respiration, maximal respiration, and ATP link respiration. Canagliflozin treatment (CANA) notably enhanced these mitochondrial functions (Figure 1C–G).

Additionally, the high-glucose condition escalated mitochondria-derived reactive oxygen species (ROS) levels compared with the normoglycemic group (control), an effect countered by canagliflozin (Figure 1H,I). Depolarization of the mitochondrial membrane potential (MMP) indicates mitochondrial dysfunction, which consistently triggers cellular apoptosis. In the HG group, decreased MMP and increased apoptosis were observed (Figure 1J,L,M). Correspondingly, ATP content, a direct measure of mitochondrial function, was significantly reduced in the HG group but was restored by canagliflozin (Figure 1K).

Exploring mitochondrial dysfunction further, western blot analysis showed increased expression of mitochondrial membrane proteins (TOM20 and Cytochrome C Oxidase IV, COXIV) in the high-glucose environment (Figure 1N–P). This was corroborated by elevated mitochondrial DNA (mtDNA) levels (Figure 1Q), suggesting an increase in mitochondrial quantity, which normalized post-canagliflozin treatment.

These experiments underscore that high-glucose conditions, reflective of diabetes mellitus, precipitate mitochondrial dysfunction characterized by impaired respiratory capacity and ATP synthesis, alongside an increase in mitochondrial quantity. Canagliflozin’s mitigative effects on these parameters provide compelling evidence of its potential in addressing mitochondrial dysfunction in diabetic environments.

### 2.2. Canagliflozin Effectively Restored Impaired PINK1-Parkin-Dependent Mitophagy in High-Glucose-Treated H9C2 Cells

**Figure 2:**
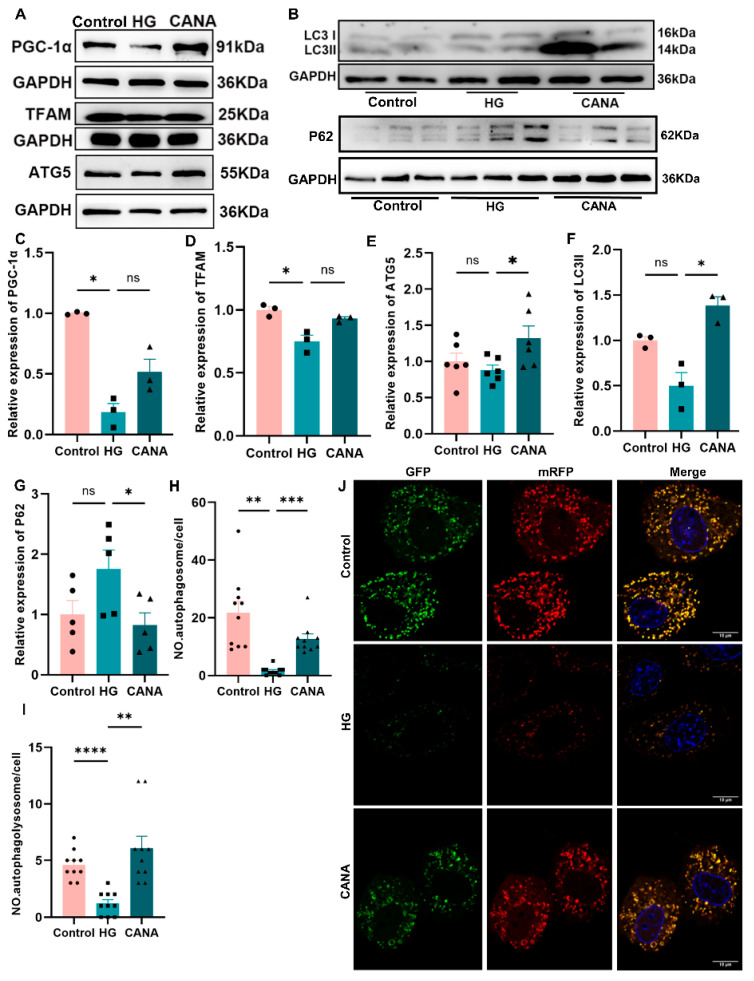
Canagliflozin effectively restored impaired PINK1-Parkin-dependent mitophagy in high-glucose-treated H9C2 cells. (**A**–**G**) Western blot for PGC-1α, TFAM, LC3, P62, and ATG5; all protein expressions were normalized to GAPDH protein expression. For P62, used Hanks’ balanced salt solution to simulate a starvation environment in the last 4 h of the intervention period. (**H**–**J**) Tandem fluorescent mRFP-GFP-LC3 adenovirus was used to detect autophagic flux; scale bar: 10 μm; original magnification: ×100. Quantification of autophagosomes (yellow puncta) and autophagolysosomes (red puncta); each groupcounted 10 cells. Control, 5.5 mmol/L glucose; HG, 60 mmol/L glucose; CANA, HG + 20 μmol/L canagliflozin. All data are shown as mean ± SEM. * *p* < 0.05; ** *p* < 0.01; *** *p* < 0.001; and **** *p* < 0.0001; ns, no significance.

In exploring the causes behind the observed alterations in mitochondrial quantity under high-glucose conditions, we focused on the dual processes of mitochondrial biogenesis and clearance. Mitochondrial biogenesis is primarily governed by the control of mitochondrial transcription regulators, namely, peroxisome proliferator-activated receptor γ coactivator-1 alpha (PGC-1α) and mitochondrial transcription factor A (TFAM). Western blot analysis showed TFAM and PGC-1α were significantly reduced in the HG group, while they were restored in the CANA group (Figure 2A,C,D). However, there was no difference in the RNA levels of TFAM among these groups (Supplementary Figure S1A).

On the mitophagy front, the autophagic marker microtubule-associated protein 1 light chain 3 (LC3) undergoes a transformation from LC3-I to LC3-II during autophagy activation, marking autophagosome formation. In this study, an increase in the LC3-II/LC3-I ratio was observed in the CANA group, indicating enhanced autophagic activity (Figure 2B,F). Conversely, the accumulation of sequestosome 1 (SQSTM1/p62), an autophagy substrate, is indicative of autophagy inhibition. Our findings showed a decrease in p62 levels in both the normoglycemic and CANA-treated groups under autophagy-inducing conditions, such as starvation, confirming the activation of autophagic flux, which was not observed in the HG group (Figure 2B,G). Similarly, canagliflozin treatment significantly increased the expression of autophagy-related 5 (ATG5), a critical protein in autophagy (Figure 2A,E). To further elucidate the autophagic flux, we employed an mRFP-GFP-LC3 double-labeled adenovirus for cellular infection. This analysis demonstrated a decrease in both autophagosomes and autolysosomes in the HG group, a trend that was reversed by canagliflozin, indicating a restoration of autophagic processes (Figure 2H–J).

**Figure 3:**
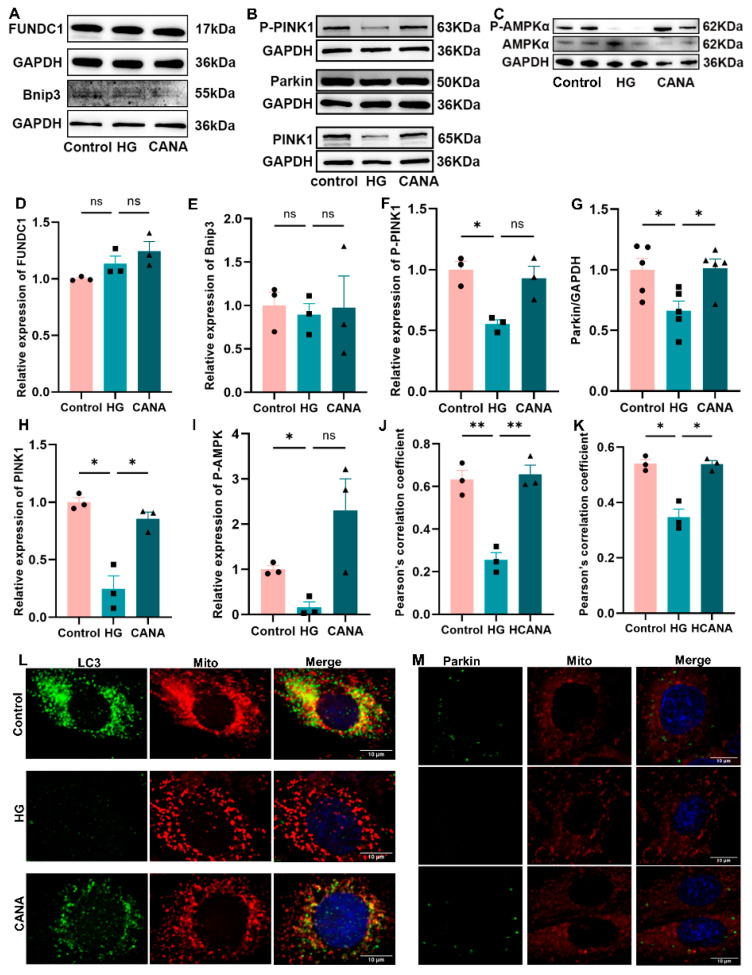
Canagliflozin effectively restored impaired PINK1-Parkin-dependent mitophagy in high-glucose-treated H9C2 cells. (**A**–**I**) Western blot for FUNDC1, Bnip3, Parkin, PINK1, P-PINK1, and P-AMPK proteins. All protein expressions were normalized to GAPDH protein expression. (**J**) Pearson’s correlation coefficient for co-localization of LC3 and mitochondria using ImageJ 2 software. (**K**) Pearson’s correlation coefficient for co-localization of Parkin and mitochondria using ImageJ 2 software. (**L**) Co-localization of LC3 puncta with mitochondria assessment using double immunofluorescence with LC3 antibody and MitoTracker Red; scale bar: 10 μm; original magnification: ×100. (**M**) Co-localization of Parkin puncta with mitochondria assessment using double immunofluorescence with Parkin antibody and MitoTracker Red; scale bar: 10 μm; original magnification: ×100. Control, 5.5 mmol/L glucose; HG, 60 mmol/L glucose; CANA, HG + 20 μmol/L canagliflozin. All data are shown as mean ± SEM. * *p* < 0.05; ** *p* < 0.01; ns, no significance.

Mitophagy primarily comprises two well-established pathways: the ubiquitin-mediated PINK1-Parkin pathway and the receptor-mediated pathway (involving Bcl-2/adenovirus E1B19kDa interacting protein 3 [Bnip3] or FUNDC1). We first assessed the RNA levels of Bnip3, FUNDC1, and PINK1. Despite no significant differences across the groups for Bnip3, FUNDC1, and PINK1 RNA levels, PINK1 expression exhibited a downward trend in the HG group, which canagliflozin administration effectively reversed (Supplementary Figure S1B–D). Protein expression levels of Bnip3 and FUNDC1 remained unchanged across all groups (Figure 3A,D,E). Notably, PINK1, Parkin, and phosphorylated PINK1 (Ser228) expression were significantly reduced under HG conditions, a trend counteracted by canagliflozin (Figure 3B,F–H).

Parkin, LC3, and MitoTracker were used to assess the extent of PINK1-Parkin-dependent mitophagy. HG conditions diminished LC3 expression and mitochondrial co-localization, effects markedly reversed by canagliflozin (Figure 3J,L). A similar situation occurred in Parkin expression and mitochondrial co-localization (Figure 3K,M). Qualitative assessments also confirmed the activation of autophagy and mitophagy by canagliflozin (Supplementary Figure S1E–J).

Additional investigations were conducted to ascertain the potential alterations occurring upstream of autophagy. AMPK phosphorylation, which is a pivotal determinant in the upstream regulation of autophagy, notably decreased in the HG group, whereas canagliflozin administration restored its expression level (Figure 3C,I).

These findings delineate how high glucose disrupts mitochondrial biogenesis and mitophagy, impairments that canagliflozin effectively ameliorates, likely through increasing the phosphorylation of AMPK, offering insights into its potential mechanistic actions against diabetic complications.

### 2.3. Canagliflozin Improved Cardiac Dysfunction and Metabolic Abnormalities in Diabetic Mice

**Figure 4:**
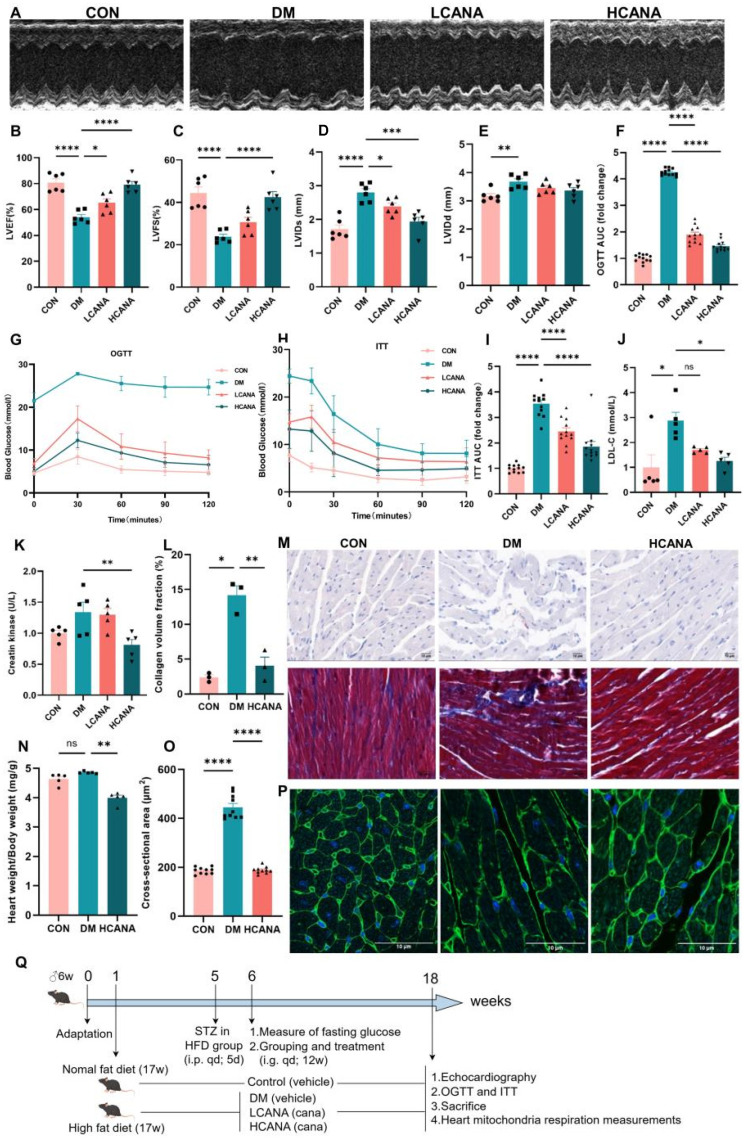
Canagliflozin improved cardiac dysfunction and metabolism abnormalities in diabetic mice. (**A**) Representative echocardiographic analysis showing M-mode images. (**B**–**E**) LVEF, left ventricle ejection fraction; LVFS, left ventricle fractional shortening; LVIDd, left ventricle diastolic internal diameter; LVIDs, left ventricle systolic internal diameter (*n* = 6). (**F**–**I**) Blood glucose levels in OGTT, ITT, and their AUC; *n* = 12. (**J**,**K**) Plasma levels of creatine kinase and low-density lipoprotein cholesterol (*n* = 5). (**L**) Collagen deposition quantitatively analyzed as collagen volume fraction (%) using ImageJ 2 software. (**M**) Oil Red O and Masson’s trichrome staining, respectively; scale bar: 10/20 μm; original magnification: ×400. (**N**) The ratio of heart weight/body weight (*n* = 5). (**O**) Quantitative analysis of cardiomyocyte cross-sectional area. (**P**) Cardiac myocyte membrane representative immunohistochemical images of wheat germ agglutinin staining; scale bar: 10 μm; original magnification: ×63. (**Q**) Flow chart of animal experiments. Control, normal control mice; DM, diabetic mice; LCANA, 10 mg/kg canagliflozin; HCANA, 30 mg/kg canagliflozin; i.p., intraperitoneal injection; i.g., by gavage; qd, once daily; HFD, high-fat diet. All data are shown as mean ± SEM. * *p* < 0.05; ** *p* < 0.01; *** *p* < 0.001; and **** *p* < 0.0001; ns, no significance.

The process of the animal experiments is shown in Figure 4Q. The cardioprotective function of canagliflozin was further investigated in a diabetic mouse model. Cardiac function, assessed through echocardiography, revealed significant impairments in left ventricular ejection fraction (LVEF) and left ventricular fractional shortening (LVFS) among diabetic mice (DM), alongside increases in left ventricular internal diameter during diastole (LVIDd) and systole (LVIDs) (Figure 4A–E). A high dose of canagliflozin (HCANA) significantly enhanced the above-mentioned cardiac markers to a greater extent than a low dose (LCANA).

Glucose metabolism was further evaluated via the oral glucose tolerance test (OGTT) and insulin tolerance test (ITT), with differences quantified by the area under the curve (AUC). Diabetic mice demonstrated significant deficits in glucose tolerance and insulin sensitivity, which were ameliorated by canagliflozin, particularly at higher dosages (Figure 4F–I). Simultaneously, plasma creatine kinase and LDL-C levels increased in diabetic mice. Notably, a high dose of canagliflozin was more effective than a low dose in restoring these indicators (Figure 4J,K). Collectively, these outcomes underscore the superior therapeutic benefits of high-dose canagliflozin in improving cardiac function and metabolic parameters in diabetic mice, guiding the focus of our subsequent investigations towards this dosage group.

Masson’s trichrome and Oil Red O staining were employed to assess collagen deposition and lipid accumulation, respectively, within myocardial tissues. Collagen and lipid deposition in diabetic mice increased and significantly diminished following canagliflozin administration (Figure 4L,M). The size of the cardiomyocytes was assessed using wheat germ agglutinin (WGA) staining, which revealed a significant increase in the cross-sectional area in diabetic mice. However, canagliflozin effectively ameliorated these effects (Figure 4O,P). Moreover, canagliflozin treatment succeeded in normalizing the heart-to-body weight ratio index, further demonstrating its efficacy in mitigating cardiac alterations associated with diabetes (Figure 4N).

### 2.4. Cardiac Tissue Proteomics Analysis of Canagliflozin-Treated Mice

**Figure 5:**
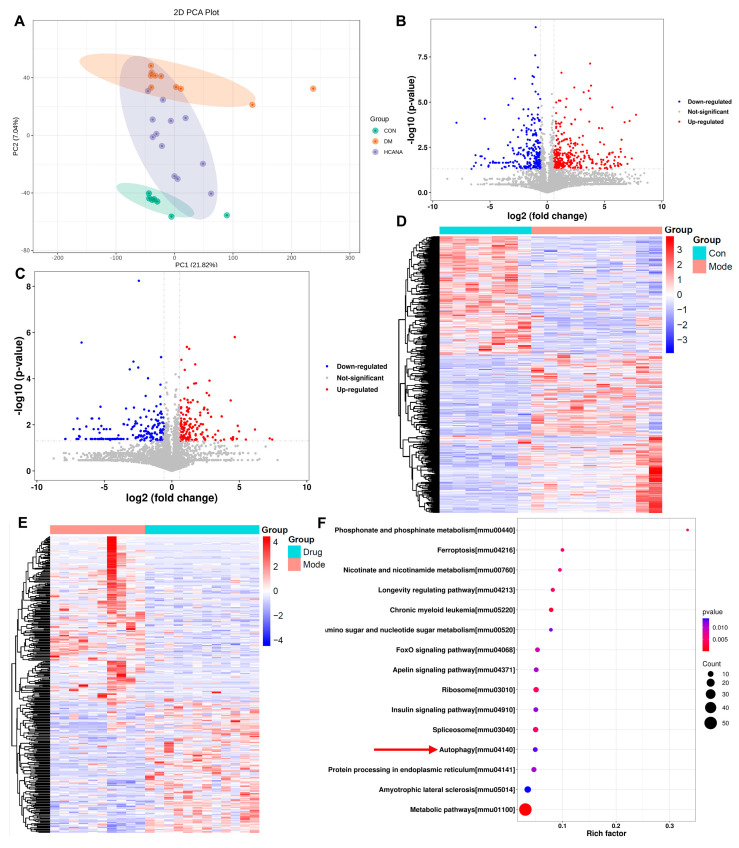
Cardiac tissue proteomics analysis of canagliflozin-treated mice. (**A**) Principal component analysis of three groups. (**B**) Volcano maps of differentially expressed proteins between CON and DM groups. (**C**) Volcano maps of differentially expressed proteins between DM and HCANA groups. (**D**,**E**) Heat maps and hierarchical cluster analysis of DEPs in CON vs. DM and DM vs. HCANA. (**F**) KEGG enrichment pathway. CON, normal control mice; DM, diabetic mice; HCANA, 30 mg/kg canagliflozin.

In an extensive label-free quantitative proteomics analysis of heart tissues from control (CON), diabetes mellitus (DM), and high-dose canagliflozin-treated (HCANA) mice, we identified a total of 4448 proteins. Principal component analysis (PCA) underscored significant variations in protein expression profiles among the groups, illustrating distinct proteomic alterations induced by DM and modulated by HCANA treatment (Figure 5A). A total of 573 and 338 differentially expressed proteins (DEPs) were identified between CON vs. DM and DM vs. HCANA comparisons, respectively, based on the criteria of Student’s *t* test *p* < 0.05 and fold change ≤ 0.67 or fold change ≥ 1.5 (Supplementary Table S2). Subsequently, we visualized the DEPs through the creation of volcano maps (Figure 5B,C). In comparison to the CON, the DM group exhibited 314 upregulated proteins and 259 downregulated proteins. Likewise, the HCANA group showed 150 upregulated proteins and 188 downregulated proteins, in contrast to the DM group. Hierarchical clustering and heat map visualization of these DEPs revealed distinct expression patterns, particularly between DM and HCANA groups, indicating canagliflozin’s effect on the proteomic profile (Figure 5D,E). A Kyoto Encyclopedia of Genes and Genomes (KEGG) pathway enrichment analysis of the HCANA group’s DEPs underscored the modulation of metabolic and autophagy pathways, pointing to a comprehensive impact on cellular processes (Figure 5F). This is further supported by an observed increase in LC3 levels, a hallmark of autophagy activation, upon canagliflozin treatment. Additionally, the Gene Ontology (GO) analysis indicated a significant upregulation in mitochondrial gene expression and translation (Supplementary Figure S1Q). Collectively, these proteomic insights reveal canagliflozin’s potential to mitigate DM-induced alterations by improving mitochondrial function and promoting autophagy, underscoring its therapeutic promise for diabetic cardiomyopathy.

### 2.5. Canagliflozin Resulted in a Substantial Enhancement of Cardiac Mitochondrial Quality in Diabetic Mice

**Figure 6:**
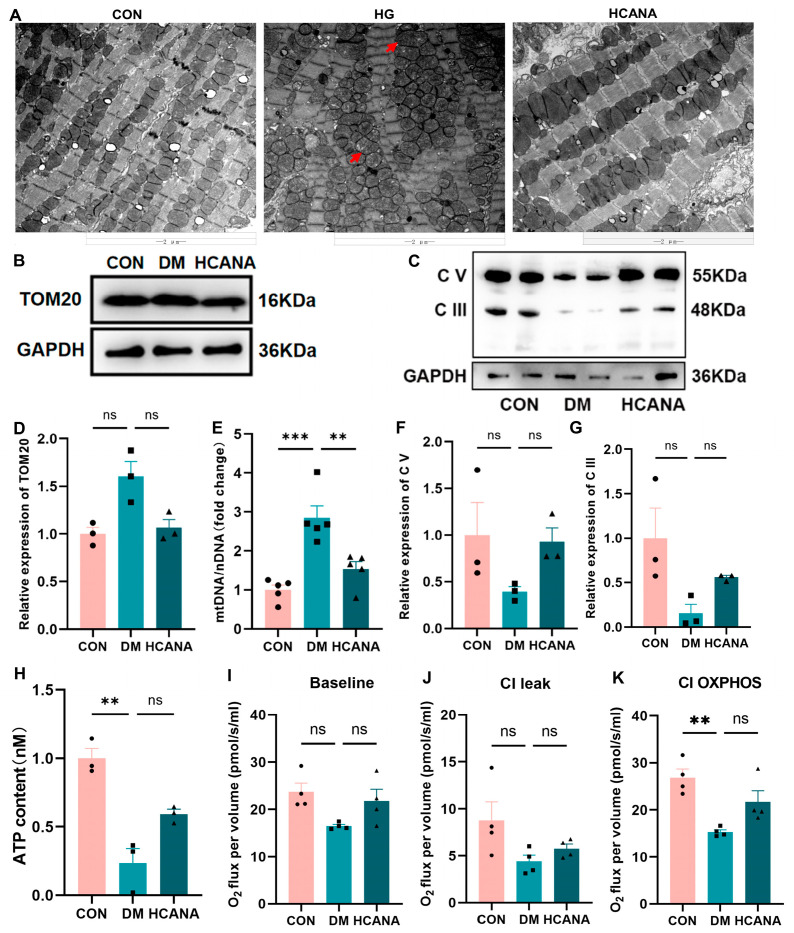
Canagliflozin resulted in a substantial enhancement in cardiac mitochondrial quality in diabetic mice. (**A**) Transmission electron microscopy of the heart tissue mitochondrial structure in each group; red arrows, damaged mitochondria; original magnification: ×10,000. (**B**–**D**,**F**,**G**) Western blot for TOM20 and mitochondrial respiration complexes proteins in heart tissue (GAPDH internal reference). C V and III: mitochondrial respiratory chain complexes V and III. (**E**) The ratio of mitochondrial-encoded DNA to nuclear-encoded (*n* = 5). (**H**) Heart tissue ATP content determination using ATP assay kit (*n* = 3). (**I**–**K**) Mouse heart mitochondria high-resolution respirometry was measured using an Oroboros 2k instrument (*n* = 4). All data are shown as mean ± SEM. ** *p* < 0.01; *** *p* < 0.001; and ns, no significance.

Transmission electron microscopy (TEM) analysis provided a stark visualization of the myocardial tissue disarray in diabetic mice, characterized by a disorganized and crowded mitochondrial arrangement and structural damage, such as vacuolation and mitochondrial cristae disruption (Figure 6A). This pathological state was accompanied by an increase in mitochondrial quantity, as evidenced by elevated mtDNA copy numbers and TOM20 protein expression levels. High-dose canagliflozin treatment not only normalized the mitochondrial count but also ameliorated the structural anomalies, restoring orderly mitochondrial arrangement and integrity (Figure 6B,D,E).

Subsequently, the expression of the mitochondrial respiratory complex showed that complexes V and III decreased in the DM group and were reversed upon canagliflozin administration (Figure 6C,F,G). This restoration aligns with observed improvements in ATP levels within myocardial tissues, which were significantly reduced in diabetic conditions(Figure 6H). Moreover, evaluating mitochondrial respiratory function using Oroboros 2k revealed compromised performance across three respiratory states in cardiomyocyte mitochondria from diabetic mice. Intriguingly, canagliflozin administration demonstrated a capacity to enhance these respiratory states, pointing towards a significant amelioration of mitochondrial functionality (Figure 6I–K).

These findings collectively underscore the profound impact of high-dose canagliflozin in counteracting diabetes-induced mitochondrial and cardiac dysfunction. Through structural restoration and functional improvement of mitochondria, canagliflozin emerges as a potent therapeutic agent for mitigating diabetic cardiomyopathy, highlighting its dual action on both mitochondrial quantity and quality.

### 2.6. Canagliflozin Activated PINK1-Parkin-Dependent Mitophagy in Diabetic Mice

**Figure 7:**
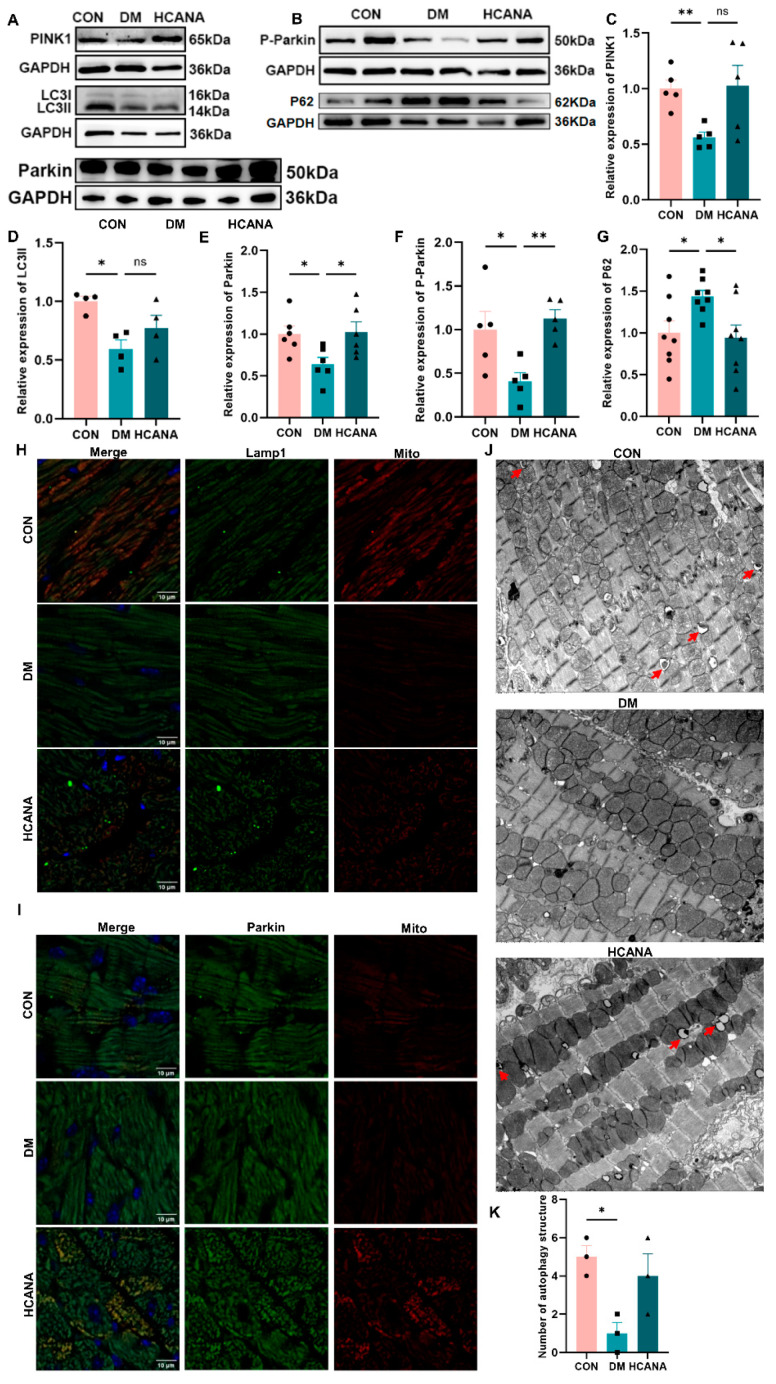
Canagliflozin activated PINK1-Parkin-dependent mitophagy in diabetic mice. (**A**–**G**) Western blot for Parkin, PINK1, P-Parkin, P62, and LC3 proteins of the heart tissue (GAPDH internal reference). (**H**) The co-localization analysis of mitochondria and lysosomes with TOM20 and Lamp1 antibodies in the heart tissue examination using a confocal microscope; scale bar: 10 μm; original magnification: ×100. (**I**) The co-localization analysis of mitochondria and Parkin with TOM20 and Parkin antibodies examination using a confocal microscope in the heart tissue; scale bar: 10 μm; original magnification: ×100. (**J**) Transmission electron microscopy in the heart tissue in each group; red arrows, autophagolysosome or autophagosome; original magnification: ×10,000. (**K**) Quantitative analysis of autophagosome, autophagolysosome, and mitophagosome (*n* = 3). CON, normal control mice; DM, diabetic mice; HCANA, 30 mg/kg canagliflozin. All data are shown as mean ± SEM. * *p* < 0.05; ** *p* < 0.01; ns, no significance.

In assessing PINK1-Parkin-dependent mitophagy within myocardial tissues, we observed a significant downregulation of PINK1 and Parkin protein levels in the DM group. Canagliflozin treatment notably restored these key proteins’ expression levels (Figure 7A,C,E). This restoration was accompanied by an increase in LC3II expression and a decrease in P62, suggesting enhanced autophagic flux (Figure 7A,B,D,G). The phosphorylation of Parkin at serine 65 by PINK1, essential for initiating mitophagy, was reduced in DM but was effectively reinstated following canagliflozin administration (Figure 7B,F).

Further examination through co-localization studies revealed diminished associations between mitochondria and Parkin, as well as between mitochondria and lysosomes, in diabetic conditions. These interactions, indicative of mitophagic activity, were significantly enhanced with canagliflozin treatment, suggesting improved mitophagy (Figure 7H,I). The relevant qualitative analysis is in Supplementary Figure S1K–P. Transmission electron microscopy provided a direct visualization of the mitophagic process. In diabetic myocardial tissue, the presence of autophagolysosomes and autophagosomes was markedly scarce, contrasting with the considerable increase in these autophagic structures following canagliflozin intervention (Figure 7J,K).

### 2.7. Canagliflozin Failed to Enhance Mitochondrial Function and Mitophagy in the Absence of PINK1 in High-Glucose-Treated Cells

**Figure 8:**
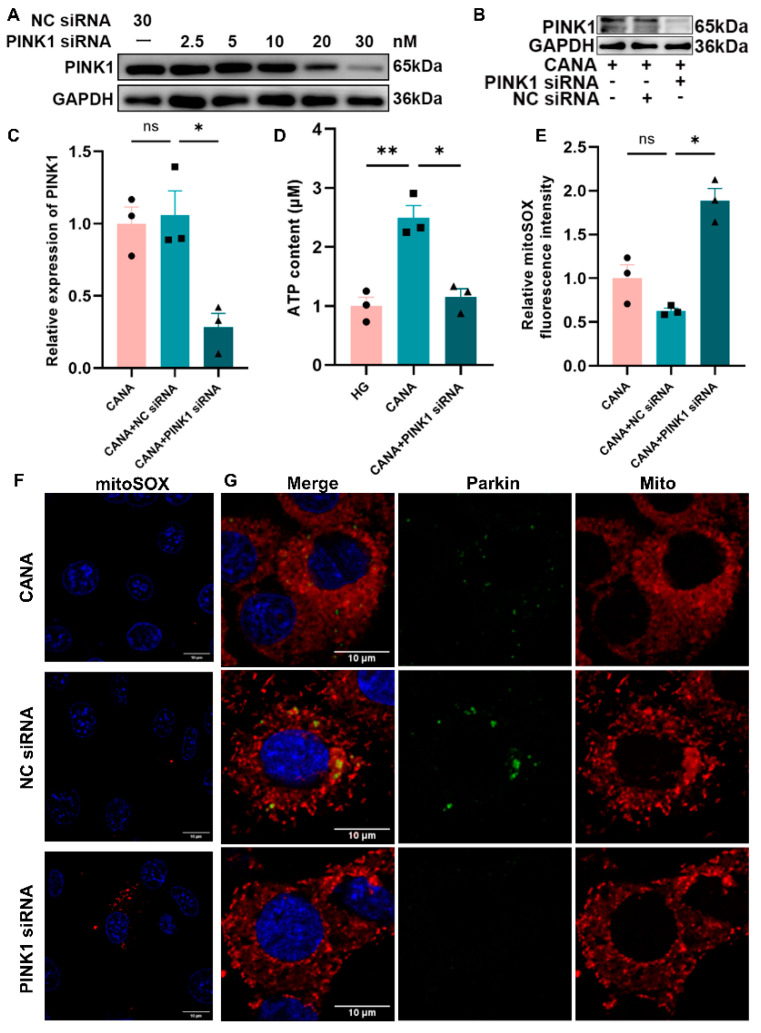
Canagliflozin failed to enhance mitochondrial function and mitophagy in the absence of PINK1 in high-glucose-treated cells. (**A**) Western blot for PINK1 protein in H9C2 cells following siRNA transfer for 48 h (GAPDH internal reference, NC siRNA: negative control siRNA). (**B**,**C**) Western blot for PINK1 protein in H9C2 cells following siRNA transfer and canagliflozin administration for 48 h. (**D**) ATP content determination using ATP assay kit in H9C2 cells following siRNA transfer for 48 h (*n* = 3). (**E**) Relative mean fluorescence intensity of MitoSOX (Mean = IntDen/Area). (**F**) Mitochondrial reactive oxygen species detection using MitoSOX (red); scale bar: 20 μm; original magnification: ×100. (**G**) Co-localization analysis of mitochondria and Parkin with MitoTracker and Parkin antibody examination using a confocal microscope; scale bar: 10 μm; original magnification: ×100. All data are shown as mean ± SEM. * *p* < 0.05; ** *p* < 0.01; ns, no significance.

In our study, the essential role of PINK1−Parkin-dependent mitophagy in maintaining mitochondrial functionality in cardiomyocytes was further elucidated through targeted depletion of PINK1 using small interfering RNA (siRNA). An optimal siRNA concentration of 30 nM was identified, which significantly knocked down PINK1 expression levels in H9C2 cells (Figure 8A). Western blotting confirmed the reduction in PINK1 levels by siRNA treatment compared to controls, underscoring the specificity of the siRNA effect (Figure 8B,C). The absence of PINK1 markedly inhibited the capacity of canagliflozin to counteract the adverse effects of high glucose on mitochondrial function. This was demonstrated by the inability to restore ATP and mitochondrial ROS to normal levels in the absence of PINK1, highlighting the necessity of PINK1 for canagliflozin’s protective mechanism against mitochondrial dysfunction induced by high glucose (Figure 8D–F). Conversely, in the presence of PINK1, canagliflozin effectively normalized mitochondrial function, emphasizing the critical role of the PINK1−Parkin pathway in mediating canagliflozin’s therapeutic effects. Further analysis investigated the interaction between PINK1 and Parkin. A decrease in PINK1 expression not only reduced Parkin levels but also adversely affected their mitochondrial co-localization, indicating a disrupted mitophagic process (Figure 8G). This suggests that the PINK1-Parkin pathway is essential for canagliflozin’s mechanism of action in mitigating high-glucose-induced mitochondrial impairment.

## 3. Discussion

This study unveils novel insights into canagliflozin’s efficacy in ameliorating mitochondrial dysfunction and enhancing cardiac function in both in vivo and in vitro models of diabetic cardiomyopathy (DCM). Our research demonstrates that hyperglycemia-induced mitochondrial dysfunction, especially characterized by impaired PINK1-Parkin-dependent mitophagy, is significantly mitigated by canagliflozin treatment. These findings underscore the drug’s potential in stimulating mitochondrial quality control mechanisms, thus offering cardioprotection in the context of diabetes.

T2DM is characterized by mitochondrial dysfunction, increased ROS production, and low ATP levels [21]; moreover, substantial evidence has indicated mitochondrial dysfunction also promote the development of DCMs [22,23,24]. Canagliflozin, traditionally known as an SGLT2 inhibitor targeting hyperglycemia, has been previously shown to preserve mitochondrial function in renal disease by restoring mitochondrial membrane potential, reducing mitochondrial ROS output, and normalizing activity across mitochondrial respiratory complexes [25]. This study broadens the understanding of canagliflozin’s therapeutic potential in heart disease.

Mitophagy, especially the PINK1-Parkin-dependent pathway, plays an indispensable role in maintaining mitochondrial and cardiac functions. Prior research has consistently shown this pathway’s inhibition in DCM, leading to aggravated mitochondrial dysfunction and exacerbated myocardial injury in diabetes [26,27,28]. This is in line with what was observed in this study. Conversely, the activation of mitophagy has been associated with improved mitochondrial functionality and enhanced cardiac performance, reinforcing the potential of targeting this pathway for therapeutic intervention in DCM [29]. Further, Alisporivir, JQ1 (BET bromine domain inhibitor) and hydrogen sulfide have been shown to increase the expression levels of PINK1 and Parkin in diabetic hearts to activate mitochondrial autophagy against DCM [28,30,31]. Recent evidence suggests that canagliflozin activates AMPK-mediated autophagy in renal tubular cells to protect them from cisplatin damage [32], or enhance macrophage autophagy to inhibit atherosclerosis progression [33]. These findings support the ability of canagliflozin to regulate autophagy. Despite these studies, no studies have looked at the effects of canagliflozin on PINK1-Parkin-dependent mitophagy in DCM or other diseases. This is also the starting point and innovation of this study. However, a computer model predicts that canagliflozin promotes the activation of the PINK1-Parkin pathway by inhibiting ubiquitin-specific proteinase 30 [34]. This gives us confidence in the results of this study. In this study, we not only confirmed the role of mitochondrial dysfunction in DCM, but also found the importance of mitophagy, especially the PINK1-Parkin pathway, which is also expected to be a new target for the treatment of multiple complications of diabetes.

Additionally, this study revealed that a high glucose concentration impeded mitochondrial biogenesis, possibly via the PGC-1α-TFAM signaling pathway. The association between impaired mitochondrial biogenesis and DCM is well documented, with reduced expression of pivotal regulators like PGC-1α and TFAM being linked to the condition, thus pinpointing them as viable therapeutic targets [14,35,36,37]. Importantly, our results extend the therapeutic narrative of canagliflozin, this aligns with recent findings that canagliflozin upregulates PGC-1α to promote biogenesis and improve mitochondrial function in obesity and adipocytes, with beneficial effects beyond the regulation of glucose [38,39]. This dual mechanism of action addresses both the mitochondrial and energetic imbalances at the heart of DCM’s pathogenesis, positioning canagliflozin as a multifaceted therapeutic agent in the management of diabetic cardiovascular complications.

This study, while providing insightful findings on canagliflozin’s impact on diabetic cardiomyopathy, acknowledges several limitations. Primarily, the use of an HFD-streptozotocin-induced T2DM mouse model might not fully capture the clinical complexities of T2DM in humans, due to the absence of T2DM-associated genetic factors. Additionally, our research was conducted exclusively with male mice to control for estrogen’s potential effects on insulin resistance, acknowledging that sexual dimorphism could influence the outcomes. This aspect highlights the need for further investigation using female models to fully understand the sex-dependent effects of canagliflozin in diabetic cardiomyopathy.

**Figure 9:**
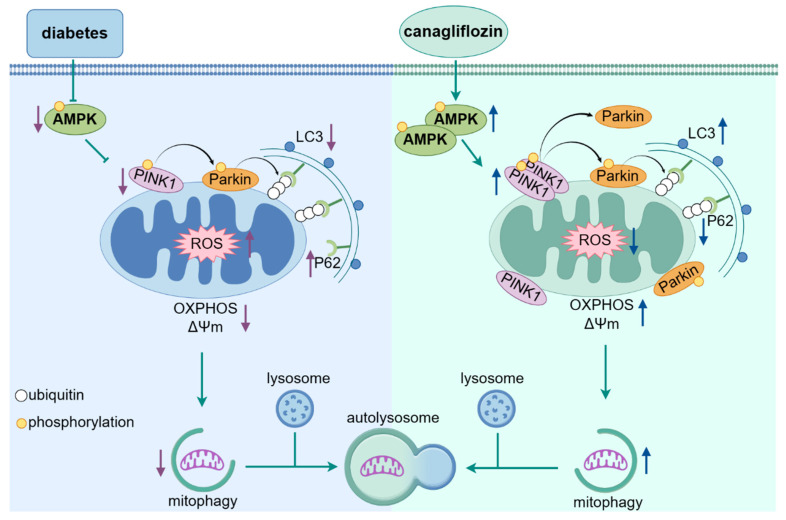
The molecular mechanism of the improvement of canagliflozin in diabetic cardiomyopathy. In diabetes, canagliflozin increases phosphorylation of AMPK, upregulates the expression of PINK1 and P-PINK1, and then, recruits and phosphorylates more Parkin, activates mitophagy, and improves mitochondrial function (oxidative phosphorylation and membrane potential recovery, ROS level decrease). OXPHOS, mitochondrial oxidative phosphorylation; ΔΨm, mitochondrial membrane potential. Figure created by Figdraw (2.0).

In summary, our results indicate that the use of canagliflozin can significantly and safely improve metabolic status and mitochondrial function as well as cardiac function. Canagliflozin treatment enhanced PINK1-Parkin-dependent mitophagy and mitochondrial biogenesis, which further elucidates the underlying mechanism. These findings provide new insights into understanding the cardioprotective properties of canagliflozin, thereby warranting additional clinical investigations (Figure 9 shows this mechanism).

## 4. Materials and Methods

### 4.1. Animal Experimental Design and Treatment

Six-week-old male C57BL/6J mice were obtained from Weitong Lihua Limited Company (Beijing, China). A total of 48 mice were obtained. After one week of adaptation, 12 mice were assigned to the normal control group and were maintained on a normal-fat diet, while the remaining were fed a high-fat diet (HFD) (60% kcal fat, Research Diets, New Brunswick, NJ, USA). Diabetes was induced through consecutive intraperitoneal injections of streptozotocin (Sigma-Aldrich, St. Louis, MO, USA, 50 mg/kg) dissolved in 0.1 mol/L citrate buffer (pH 4.5) for 5 days after 4 weeks of feeding. Normal control mice were injected with buffer alone. At the end of induction, the mice with fasting glycemia levels ≥11.1 mM, assessed using mouse tail vein blood, were considered successful T2DM models. Following diabetes confirmation, the mice were administered the vehicle or canagliflozin (#S2760, Selleck, Houston, TX, USA) once a day by gavage for another 12 weeks. The animals were categorized into the following groups: vehicle-administered normal control mice (CON, *n* = 12), vehicle-administered diabetic mice (DM, *n* = 12), low-dose canagliflozin-administered diabetic mice (LCANA, 10 mg·kg^−1^·day^−1^, *n* = 12), and high-dose canagliflozin-administered diabetic mice (HCANA, 30 mg·kg^−1^·day^−1^, *n* = 12). All mice were maintained under controlled conditions (temperature: 22 ± 2 °C, humidity: 55 ± 10%, and a 12 h light/dark cycle).

### 4.2. Cell Culture and Treatment

H9C2 cells were purchased from Procell Life Science & Technology Co., Ltd. (Wuhan, China). First, the cells were cultured in Dulbecco’s modified Eagle medium (Gibco, Grand Island, NY, USA) and incubated in 5% CO_2_ at 37 °C in a humidified atmosphere. Cells were cultured overnight, the medium was replaced. H9C2 cells were subjected to normal glucose (5.5 mmol/L, control) or high-glucose (60 mmol/L, HG) challenge for 48 h with the vehicle or canagliflozin supplement (20 μmol/L, CANA) (#S2760, Selleck, Houston, TX, USA). The H9C2 cells at passages 3–10 were used in the experimental protocols.

### 4.3. Proteomics Analysis

Total protein was extracted from the heart tissues from the control (*n* = 7), diabetes (*n* = 10), and high-dose canagliflozin-treated groups (*n* = 12), quantified, and stored at −80 °C. Proteomics sequencing and analysis were conducted by Biotree Biotech Company (Shanghai, China). Briefly, the extracted proteins were first quantified by the BCA assay, precipitated using acetone, and then, subjected to reduction, alkylation, digestion, TMT labeling, SDC cleanup, peptide desalting, and high-pH pre-fractionation. For each sample, 200 ng of total peptides was separated and analyzed with a nano-UPLC (nanoElute2) coupled to a timsTOF Pro2 instrument (Bruker) with a nano-electrospray ion source. Separation was performed using a reversed-phase column (PePSep C18, 1.9 μM, 75 μM × 25 cm, Bruker, Karlsruhe, Germany). The mobile phases were H_2_O with 0.1% FA (phase A) and CAN with 0.1% FA (phase B). Separation of samples was executed with a 60 min gradient at a 300 nL/min flow rate. Gradient B: 2% for 0 min, 2–22% for 45 min, 22–37% for 5 min, 37–80% for 5 min, 80% for 5 min. The mass spectrometer adopted DDA PaSEF mode for DDA data acquisition, and the scanning range was from 100 to 1700 *m*/*z* for MS1. During PASEF MS/MS scanning, the impact energy increased linearly with ion mobility, from 20 eV (1/K0 = 0.6 Vs/cm^2^) to 59 eV (1/K0 = 1.6 Vs/cm^2^).

The data were processed, including principal component analysis (PCA), differentially expressed protein (DEP) screening, volcano map analysis, hierarchy cluster analysis of selected DEPs, Gene Ontology (GO; MF, molecular function; CC, cellular component; and BP, biological process) and Kyoto Encyclopedia of Genes and Genomes database (KEGG) pathway enrichment analysis. The screening criteria for differentially expressed proteins were *p*-value < 0.05 and fold change ≤ 0.67 or fold change ≥ 1.5.

### 4.4. Seahorse Experiments

Seahorses were used to detect mitochondrial respiratory capacity. A Seahorse XF Cell Mito Stress Test Kit (#103015-100, Agilent, Santa Clara, CA, USA) was used to directly measure the oxygen consumption rate (OCR) using an Agilent Seahorse XFe96 Extracellular Flux Analyzer (Agilent, Santa Clara, CA, USA). First, the cells (3 × 10^3^ cells/well) were seeded onto Seahorse XF cell culture 96-well microporous plates and continued to be cultured according to the above grouping conditions. Subsequently, seahorse XF calibration solution was used to hydrate the sensor probe plate at 37 °C in a CO_2_-free incubator overnight. Next, oligomycin A, FCCP, and antimycin A at final concentrations of 1.5, 2, and 0.5 μM, respectively, were added to each well, followed by the detection of OCR following the manufacturer’s instructions. The respiration before the oligomycin intervention is the basal respiration. After adding the oligomycin (an inhibitor of ATP synthase), the decreased oxygen consumption is the respiratory capacity associated with ATP synthesis. Maximal respiration was followed by the addition of FCCP (uncoupling agent, unrestricted flow of electrons through the electron transport chain). The difference between the maximal and basal respiration is the spare respiration. Finally, a mixture of rotenone (complex I inhibitor) and antimycin A (complex III inhibitor) is added, together they shut down mitochondrial respiration, and thus, the non-mitochondrial respiration can be calculated.

### 4.5. Assessment of Cell Apoptosis

An apoptosis assay was conducted on the H9C2 cells using the Annexin V-FITC Apoptosis Detection Kit (#C1062, Beyotime, Shanghai, China). Cardiomyocytes were double-stained with Annexin V-FITC and propidium iodide following the manufacturer’s protocol. Finally, images were obtained using a confocal laser scanning microscope (NIKON, Tokyo, Japan).

### 4.6. Assessment of Mitochondrial Membrane Potential (MMP)

MMP was detected using JC-1 staining (Beyotime, Shanghai, China), following the manufacturer’s instructions. When the MMP is high, JC-1 aggregates into the mitochondrial matrix to form a polymer (J-aggregate), which can produce red fluorescence. Conversely, when the MMP is low, JC-1 cannot aggregate mitochondria in the matrix, and becomes a monomer at this time, after which green fluorescence is produced. Images were obtained and analyzed using a confocal laser scanning microscope and the ImageJ 2 software, respectively.

### 4.7. Assessment of Autophagic Flux via mRFP-GFP-LC3

We transfected cultured cells with tandem fluorescent mRFP-GFP-LC3 adenovirus (MOI  =  800; Hanbio, Shanghai, China) to measure the autophagic flux of cardiomyocytes. GFP and mRFP in mRFP-GFP-LC3 adenovirus were used to label and track LC3. The GFP and mRFP expression levels were detected using a confocal microscope (NIKON, Tokyo, Japan). Red fluorescence and yellow puncta (merging of GFP and RFP signals) represent autophagolysosomes and autophagosomes, respectively. The autophagic flux was evaluated by counting spots with different colors.

### 4.8. Mouse Heart Mitochondrial Respiration Measurements

Mitochondrial respiration was measured at 37 °C in a two-chamber respirometer Oroboros 2k (O2k; Oroboros Instruments, Innsbruck, Austria). Data acquisition and analysis were performed using the DataLab software (Version 5.2, Oroboros Instruments, Innsbruck, Austria). A substrate–uncoupler–inhibitor titration (SUIT) protocol was also used. After the mice were sacrificed, about 10 mg of fresh left ventricular myocardial tissue was weighed, and a glass homogenizer was used to fully grind the tissue on ice. The homogenate was moved to the chambers of the O2k to detect the O_2_ flux after the system calibration. The respiratory oxygen flux was measured in real time and expressed as picomoles of O_2_ per second per ml of tissue. The O2k manual titration scheme is as follows: (1) baseline, measured the mitochondrial respiratory capacity in the absence of the substrate; (2) added 5 mM glutamic acid and 2 mM malic acid to measure the respiratory leakage status of mitochondrial complex 1 (CI leak); (3) added 5 mM adenosine diphosphate to measure the oxidative phosphorylation (OXPHOS) capacity of mitochondrial respiratory transport chain complex I (CI OXPHOS). And then, Cytochrome C was added at 10 μM to assess the outer mitochondrial membrane integrity.

### 4.9. Oral Glucose and Insulin Tolerance Tests

The OGTT and ITT were performed after 12 weeks of canagliflozin administration. Fasting blood glucose levels were measured from the tail vein after an overnight fasting period using a glucometer (Abbott Laboratories, Abbott Park, IL, USA). Glucose (2 g/kg) was administered by oral gavage, and blood was obtained from the tail vein before and 30, 60, 90, and 120 min after glucose administration. Following a 4 h fasting period in the ITT, the mice were intraperitoneally injected with an insulin solution (1 IU/kg). Blood glucose levels were recorded before and 15, 30, 60, 90, and 120 min after insulin injection. Finally, the area under the curve AUC was calculated, and the between-group differences were compared.

### 4.10. Echocardiography

Echocardiography was performed using the VINNO 6 LAB ultrasound system (Vinno, Suzhou, China). Mice were anesthetized for evaluation with 2% isoflurane. The two-dimensional short-axis view was used as a guide, and left ventricle M-mode tracings were obtained close to the papillary muscle. M-mode images were obtained to measure left ventricle systolic/diastole internal diameter (LVIDs)/(LVIDd), left ventricle ejection fraction (LVEF), and left ventricular fraction shortening (LVFS). The parameters were averaged over three cardiac cycles.

### 4.11. Mitochondrial ROS Level

Mitochondrial ROS were detected using the fluorescent probe MitoSOX (#M36008, Invitrogen, Carlsbad, CA, USA), following the manufacturer’s protocol, and images were obtained using a confocal laser scanning microscope and analyzed using the ImageJ 2 software.

### 4.12. Quantitative Real-Time PCR

Quantitative real-time PCR was used to assess the relative mRNA levels and mitochondrial-encoded DNA (mtDNA) content to nuclear-encoded DNA content (nDNA). Total RNA was extracted from cells or heart tissue homogenates and subsequently reverse-transcribed to cDNA using reverse transcriptase (Vazyme, Nanjing, China), following the manufacturer’s protocol. The reactions were performed using a real-time PCR system (Thermo Fisher QuantStudio 5, Waltham, MA, USA). The level of RNA/DNA was normalized by β-actin/nDNA using the 2^(−ΔΔCt)^ method. Quantitative analysis was carried out as follows: ΔCt (target gene) = Ct (target gene) − Ct (housekeeping gene). Calculated the average value of housekeeping genes in control group. ΔΔCt = ΔCt-the same average. And then, calculate the 2^(−ΔΔCt)^. Table 1 presents the primer sequences.

### 4.13. Western Blotting

Total protein from the cardiac tissues or cardiomyocytes was extracted using an RIPA buffer supplemented with protease and phosphatase inhibitors. Protein concentrations were determined using a bicinchoninic acid assay (Thermo Fisher Scientific, Waltham, MA, USA). Equal amounts (15–20 μg) of protein of each sample were separated on SDS-PAGE gels (10%) and transferred onto polyvinylidene fluoride (PVDF) membranes. After blocking with 5% skim milk at room temperature for 1 h, we incubated the membranes with primary antibodies (diluted with blocking buffer) overnight at 4 °C. Subsequently, the membranes were washed with Tris-buffered saline Tween-20 and incubated with a secondary antibody (Bioeasytech, Beijing, China) at room temperature for 1 h. The blots were detected using a chemiluminescence detection kit (Tanon, Shanghai, China). All experiments were repeated three times. The intensity values were normalized relative to glyceraldehyde-3-phosphate dehydrogenase (GAPDH) control values. Finally, the band intensity was analyzed using the ImageJ 2 software (NIH, Bethesda, MD, USA).

### 4.14. Statistical Analyses

The data from at least three independent experiments are presented as the mean ± standard error of the mean (SEM). The Shapiro–Wilk test was used for normal distribution testing. All data conformed to the normal distribution. Multiple groups were compared by one-way ANOVA analysis. Bartlett’s test was used to determine the homogeneity of variances. In case of homogeneous variances, post hoc comparisons were made with Dunnett’s test; in case of inhomogeneous variances, the Welch test was used. All figures were made by the GraphPad Prism software (version 9.0). Statistical differences were considered at *p* ˂ 0.05 (* *p* < 0.05; ** *p* < 0.01; *** *p* < 0.001; and **** *p* < 0.0001); ns, no significance.

## Figures and Tables

**Table 1:** The sequence of primers.

Gene	Forward	Reverse
Mouse β-actin	GCAGGAGTACGATGAGTCCG	ACGCAGCTCAGTAACAGTCC
Mouse Cytochrome B	GCCACCTTGACCCGATTCTTCGC	TGAACGATTGCTAGGGCCGCG
Rat β-actin	TCAGGTCATCACTATCGGCAAT	TCAGGTCATCACTATCGGCAAT
Rat Cytochrome B	GCCTCCGATTCATGTTAAGACTA	TACGCTATTCTACGCTCCATTC
Rat TFAM	TTCCAGGAGGCTAAGGATGAGTCAG	GCTTCACACTGCGACGGATGAG
Rat PINK1	ACTACCTATGCCCATCCATCTA	CTCGGTGACAGCTAAGTCATC
Rat Bnip3	CCCAGACACCACAAGATACCAACAG	GTCAGACGCCTTCCAATGTAGATCC
Rat FUNDC1	GAGAGCGATGACGAGTCTTACGAAG	CACTGTGACTGGCAACCTGTAGAAG

## Data Availability

The datasets used and analyzed during the current study are available from the corresponding author on reasonable request.

## Supplementary Materials

The supporting information can be downloaded at: https://www.mdpi.com/article/10.3390/ijms25137008/s1.
