# Supplementary material for: Canagliflozin Mitigates Diabetic Cardiomyopathy through Enhanced PINK1-Parkin Mitophagy
Source: Int J Mol Sci. 2024 Jun 26;25(13):7008. doi: 10.3390/ijms25137008 (PMC11241502; doi:10.3390/ijms25137008)
Supplement: Supplementary file 1 [file ijms-25-07008-s001.zip › ijms-3031272-supplementary.pdf]

Name	Cat No.	Company
LC3	48394	abcam
P62	5114S	Cell Signaling Technology
ATG5	12994	Cell Signaling Technology
PINK1	23274-1-AP	proteintech
Parkin	4211	Cell Signaling Technology
Phospho-PINK1 (Ser228)	TA7081	Abmart
P-Parkin (Ser65)	36866	Cell Signaling Technology
AMPKα	5831	Cell Signaling Technology
P-AMPKα	D4060	Cell Signaling Technology
FUNDC1	49240	Cell Signaling Technology
Bnip3	12396	Cell Signaling Technology
TFAM	A13552	abclonal
PGC-1α	A11971	abclonal
COXIV	11242-1-AP	proteintech
TOM20	11802-1-AP	proteintech
CoraLite488-conjugated Goat Anti-Rabbit IgG(H+L)	SA00013-2	proteintech
CoraLite594 – conjugated Goat Anti-Rabbit IgG(H+L)	SA00013-4	proteintech
GAPDH	60004-1-Ig	proteintech
Goat Anti-Mouse IgG (H&L)-HRP Conjugated	BE0102	bioeasytech
Goat Anti-Rabbit IgG (H&L)-HRP Conjugated	BE0101	bioeasytech
CoraLite® Plus 488-conjugated PARK2/Parkin Polyclonal antibody	CL488-14060	proteintech
CoraLite® Plus 488-conjugated LAMP1 Monoclonal antibody	CL488-67300	proteintech

Table S1. Name and source of antibody, in WB, we generally dilute the antibody to1000 times.

	CON vs DM	DM vs HCANA
Up	314	150
Down	259	188
Sum	573	338

Table S2. The amount of protein differentially expressed between groups of proteomics of myocardial tissue.

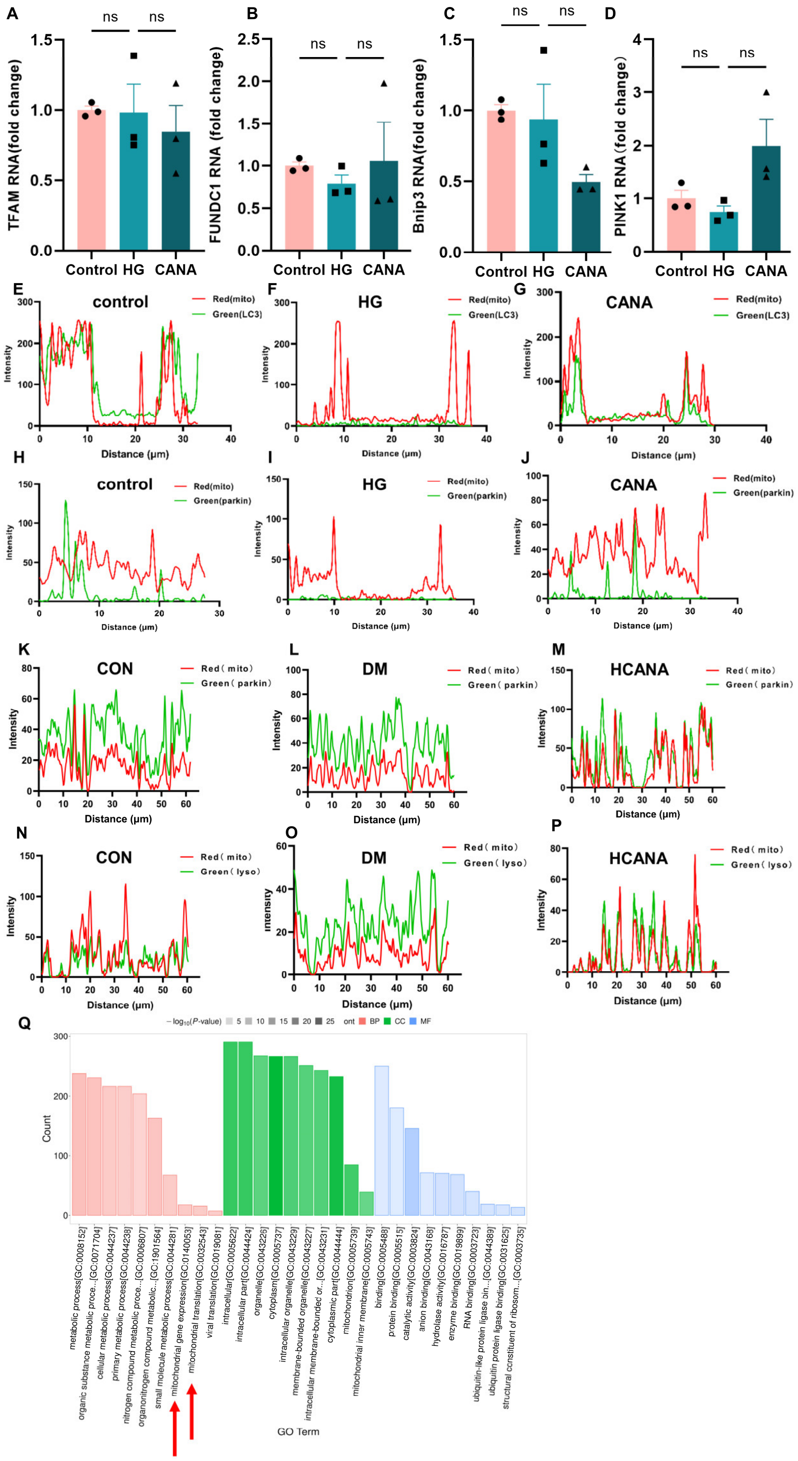

Figure S1. (A-D) The RNA level of TFAM, FUNDC1, Bnip3 and PINK1 in H9C2 cells. (E-G) Qualitative analysis of LC3 and mitochondrial fluorescence intensity in control, HG and CANA groups in H9C2 cells using ImageJ software. (H-J) Qualitative analysis of Parkin and mitochondrial fluorescence intensity in control, HG and CANA groups in H9C2 cells using ImageJ software. (K-M) Qualitative analysis of Parkin and mitochondrial fluorescence intensity in the CON, DM and HCANA groups in myocardial tissue using ImageJ software. (N-P) Qualitative analysis of lysosome and mitochondrial fluorescence intensity in the CON, DM and HCANA groups in

myocardial tissue using ImageJ software. (Q) Gene Ontology analysis of proteomics of myocardial tissue.
